# Evaluating the most effective combination of needle gauge with TENS, EMLA or topical spray for minimal pain during pediatric local anaesthesia: A randomized clinical trial

**DOI:** 10.6026/973206300221350

**Published:** 2026-03-31

**Authors:** Sai Vaishnavi Alahari, Sampath Reddy Cheruku, Ambika K Nandini, Ziauddin Mohammad, Silla Swarna Swathi, Anusha Reddy Gollampalli

**Affiliations:** 1 Department of Pedodontics and Preventive Dentistry, Sri Sai College of Dental Surgery, Vikarabad, Telangana, India;

**Keywords:** Pediatric dentistry, local anesthesia, dental anxiety, transcutaneous electric nerve stimulation (TENS), eutectic mixture of local anesthetics (EMLA)

## Abstract

Pain and anxiety during dental local anesthesia injections remain a significant challenge in pediatric dentistry, often leading to fear, poor cooperation, and negative treatment experiences in children. Therefore, it is of interest to compare the effectiveness of transcutaneous electric nerve stimulation (TENS), EMLA cream as well as conventional lignocaine topical spray, in conjunction with needles that were 25- gauge and 30-gauge for relieving anxiety and pain in children's during dental local anesthesia administration. Around 180 children ranging from 8 to 12 years old were randomly divided into six groups of equal size (n=30 for each group). The pain was evaluated via FLACC and VAS while anxiety was assessed with the Venham's Anxiety Scale along with pulse rate. The least anxiety and pain outcomes were found for the TENS + 30 gauge group, which demonstrated the longest-lasting pulse response and also the lowest score on FLACC and VAS (p less than 0.05). EMLA 30 gauge also showed less pain when compared to sprays applied to the skin, but were lower than TENS. Thus, we show using TENS that has a needle of a fine gauge as a safe, effective aid to improve the comfort of local anesthetic administration.

## Background:

Pain upon local anaesthesia (LA) injection is still one of the most powerful factors associated with dental fear and behavioural resistance in children, which consequently can affect cooperation and future dental attitude [1]. And even if treatment or surgery can be achieved painlessly under anesthetia, the injection itself is often considered the most distressing part of a prosthetic visit [2]. The modern practice of pediatric dentistry increasingly advocates multimodal approaches to minimize pain, as well as anticipatory anxiety which are clinically feasible for chairside use [3]. Choice of needle gauge has become an increasingly popular periprocedural factor as smaller-diameter needles may decrease tissue displacement and trauma, thus improving comfort perception [4]. Concurrently, topical anaesthetic agents and non- pharmacologic neuromodulation modes of intervention like transcutaneous electrical nerve stimulation (TENS) are being studied for their potential ability to attenuate nociceptive input, with the resultant perception of pain associated with injections [5]. Therefore, it is of interest to find the best combination of topical application and needle gauge with minimum pain and anxiety during LA administration in children.

## Materials and Methods:

A clinical randomized trial was conducted at the Department of Pedodontics and Preventive Dentistry, Sri Sai College of Dental Surgery, Vikarabad, India. The study was approved as ethical (IRB No. 811/SSCDS/IRB-E/2022-23) and the study was registered in the Clinical Trials Registry-India (CTRI/2023/07/055438). The study included 180 healthy children ages 8-12 years who required dental treatment in the context of LA were enrolled with parental consent. Children suffering from systemic diseases or a lack of consent were not included. Participants were randomly assigned through a lottery method to the following six categories (n=30 each) (Group IA (topical spray of lignocaine + 25G) (spray + 25G), Group IB (spray + 30G) as well as Group IIA (TENS + 25G) as well as Group IIB (TENS + 30G) Group IIIA (EMLA + 25G) as well as Group IIIB (EMLA + 30G). LA was administered with the 4% dose of articaine. The degree of pain was measured through an assessment tool called the Visual Analog Scale (VAS) and FLACC behavioral scale. Anxiety was measured with Venham's Anxiety Scale and pulse oximetry (pulse rate) measured before and after injection. The data were analysed with SPSS Version 20.0. Comparisons between groups were made with ANOVA, within-group comparisons using repeated measures ANOVA along with Bonferroni post-hoc analyses were employed and p less than 0.05 being considered statistically significant.

## Results:

The outcomes of anxiety and pain differed significant between the groups (p <0.05). The group with TENS + 30G (IIB) showed the lowest scores for pain (VAS 1.4 and FLACC 1.5) and the highest decrease of Venham anxiety ratings, as well as a minimal pulse rate fluctuations, indicating higher physiological stability. The EMLA +30G (IIIB) came in second place with lower anxiety and pain compared to spray groups. The group with spray + 25G (IA) consistently had the most negative results Table 1 (see PDF). Post-hoc testing showed that the use of TENS with a 30-gauge needle (II-B) led to better results in pain and anxiety than II-A in all treatment groups. IIIB EMLA + 30G ranked second in the scale and this revealed that comfort might be associated not only with depth of topical anesthetic but injected solution combined with mechanical-induced trauma. TENS+25G (IIA) was superior to both topical spray groups despite the thicker needle, suggesting a neuromodulating effect even with a stouter needle. Topical spray as a routine was the next least effective, especially with 25G (Table 2 - see PDF).

## Discussion:

This study was a randomized clinical trial to compare pain perception and anxiety associated with local anaesthetic injection in paediatric dentistry when using three different methods of adjuvanting (using topical lignocaine spray, EMLA cream and TENS) combined with two different needle gauges (25G and 30G). The most evident result was seen in the TENS with 30-gauge needle which resulted in better subjective (VAS), behavioural (FLACC) and physiological (pulse rate) output. These results are consistent with recent randomizedpediatric dental trials that have demonstrated how TENS-based neuromodulation, before injection, reduces procedural pain and enhances cooperative behaviour of children during local anesthesia delivery [6]. Similar recent evidence also demonstrates the efficacy of TENS and alternative non-pharmacologic sensory modulation techniques in reducing injection-related pain in children [7]. The clinical benefit of multimodal approaches is further evidenced by a recent study where several analgesic regimens were compared in pediatric local anesthesia. Randomized, placebo-controlled and open-label trials have consistently demonstrated better pain reduction with combined procedural modifications supplemented by sensory or neuromodulatory adjuncts compared to standard topical spray use alone [8]. Indeed, in the current trial, pain and anxiety scores of the topical spray groups (especially for spray + 25G) exhibited poor results, indicating that superficial topical spray alone may not provide adequate anesthetic depth to overcome injection-related nociception. This idea is further encouraged by recent evidence where more advanced topical agents such as EMLA are reported to produce enhanced pain reduction during inferior alveolar nerve block compared to conventional agents, when used optimally [9]. Of the subgroups examined on average, EMLA + 30G showed the second best performance in this study. In fact, recent randomized controlled trials have demonstrated that EMLA and other topical anesthetics do provide a statistically significant reduction in needle insertion pain for school-aged children after application with perhaps mixed results depending on the degree of mucosal thickness, saliva dilution and contact time [9]. Moreover, novel techniques like microneedle patch-based topical anesthesia have offered encouraging advancements with improvements in palatal anesthesia comfort as well and reflecting on the emerging role of topical means in pediatric pain management [10]. Nevertheless, the latest paediatric procedural pain guidelines highlight that topical anesthesia alone may not be adequate for needle-related distress and should complement behavioural or sensory modulation interventions to achieve optimal results [11]. The needle gauge was also identified as significant technical factor. In interventions, 30-gauge needles usually were found to have a better performance than their 25-gauge counter parts; in line with guideline that smaller needle diameter reduces the amount of tissue displacement and mechanical insults. Moreover, a recent systematic review comparing traditional syringe injection and computer controlled local anesthetic delivery shows that atraumatic delivery systems can even more effectively decrease pain perception and improve acceptability for children [12]. These data support that injection comfort is modulated by both pharmacological and mechanical factors. The general paediatric dentistry literature also suggests the relevance of sensory focussed adjuncts such as pre-cooling. The latter finding is in agreement with the sensory competition theory that might add to topical anesthetics as; recent *in vivo* pediatric trials have indicated that vasoconstriction of injection site greatly mitigates the amount of perceived pain elicited by LA induction [13]. Furthermore,needle-free local anesthetic techniques are also available for behavioral clinical use that can reduce pain related to restorativetreatments in children, but feasibility depends on the availability of equipment and type of office [14]. Lastly, behavioural support is still needed for pain management in children. Recent randomized controlled studies have shown that structured behaviour guidance can markedly diminish anxiety and perceived pain when administering local anesthesia, strengthening the argument forcombining behavioural techniques with pharmacologic and neuromodulatory approaches [15]. The findings from our study, on the whole, support TENS with a fine-gauge needle as an efficient, appealing and child-friendly method of reducing injection pain and anxiety in pediatric dentistry.

## Conclusion:

TENS combined with 30-gauge needle resulted in the least pain and anxiety of pediatric dental L.A. EMLA plus a 30-gauge needle was effective but always less so than TENS on all variables. "Regular" topical spray, especially using a 25G needle, was least effective and should not be used as an isolated pain-reduction method.
